# Analecta Minora, or Little Periscope

**Published:** 1825-01-01

**Authors:** 


### ANALECTA MINORA,

OR

### *LITTLE PERISCOPE.*

——— sparsa Colligens.

1. *Preservation of Dead Subjects.* Mr. Amesbury informs the Editor of the Medical Repository, that he has derived great advantage in the preservation of subjects for dissection, by using a mixture of the nitrate of potass and muriate of soda, in the proportion of one-fourth of the former to three-fourths of the latter. In the winter season, he has preserved subjects so well by the use of these salts that, at the expiration of six months, they were in nearly as good a state for dissection as when they first came into his possession. He uses about one pound of the mixed salts for every fifteen pounds of the subject to be preserved. His method is, to rub the salts upon the integuments at three or four times, at intervals of two days, and to throw a handful into the cavities of the thorax and abdomen, when he wishes to preserve the viscera. To prevent certain parts, as the fingers, toes, and places where the cuticle is abraded, from getting dry and withering, he wraps them in cloths wetted with a solution of the salts in water.

2. *Emplasto-dermic Treatment of Diseases.* Two hospital students of Paris,* have been making experiments on the introduction of various medicinal substances into the constitution, through the medium of the abraded surfaces of blisters, and other sores. In this way, it is desirable, of course, to act with substances that evince great power in a small volume. The authors of these experiments aver, that they have ascertained the *doses* of many of the principal active remedies administered according to this method. When the substances are too irritating, they incorporate them with cerates or jellies—if, on the other hand, they have a tendency to dry up the sore to which they are applied, they are to be mixed with some blistering ointment. In this way, they have calmed to sleep a considerable number of sleepless patients by the external use of acetate of morphium, as also, eased the pains of rheumatism—cured chronic catarrhs, &c. By the external application of the sulphate of quinine, they say they have stopped both tertian and quartan intermittents that resisted this powerful remedy, when taken internally. In a case of neuralgia of the temple, complicated with hysteria, the acetate of morphium and assafœtida, externally applied, effected a cure, when other remedies had failed. But the most remarkable circumstance, was a case of tetanus, produced by the external application of nux vomica to a blistered surface, and cured almost instantaneously by substituting acetate of morphium for the nux vomica. Musk, digitalis, tartar emetic, purgatives, diuretics, sudorifics, &c. have been employed in the *emplasto-dermic* manner, with (it is said) the most satisfactory results.—*Gazette de Santé*, 5 *Juillet*.

3. *Contagious Glanders in Horses.* It is said that Mr. Sewel, the very able Assistant Professor of the Veterinary College, and celebrated Neurotomist, has been so fortunate as to discover a remedy for the abovementioned malady, hitherto considered incurable. It is sulphate of copper, given in bolus or ball, in doses of from one to two ounces daily, for several weeks —*Philosoph. Mag.*

4. M. Liaubon has ascertained, (as is stated in several of the foreign journals) that a cancerous sore, if washed with an aqueous solution of common culinary salt, soon ceases to emit that peculiarly disagreeable fœtor so characteristic of this horrible disease.

5. *Non-union of Fractures.* Mr. Amesbury has recently detailed some cases in the Medical Repository, which illustrate the beneficial influence of pressure in uniting fractures which have failed in ossific union in the first instance. This pressure is made by means of a mechanical apparatus, which has already been described in this Journal, and which promises to be of essential service in these unfortunate accidents.

6. *Substitute for Castor Oil:* by C. G. Hufeland. Dr. Hufeland states that, by mixing one drop of oil of croton with an ounce of oil of poppy, a preparation is obtained which greatly resembles castor oil, in its purgative effects. A table-spoonful is an ordinary dose, and equal to the same quantity of the more expensive preparation. A great number of experiments were tried to this effect, at the Polyclinic Institution of Berlin.

7. *Congestive Fever in the Sea.* In a most notable piece of medical topography, written by the Chevallier Isoard, and published in the Medical Repository for September last, we find the following philosophic observation.

* Messrs. Lembert and Lesieur.

"The view of the sea, observed from the town of Grégoi, presents a phenomenon very worthy of attention, and which, perhaps, may have some influence *in the development of the disease which is about to engage our attention.* The waters of the sea seem elevated and suspended above the land, which may be said to be menaced with an inevitable inundation. The ships at anchor off the bar seem hovering in the air, and at a great height above the plane of the coast; and the spectator is seized with wonder and astonishment at the sight of this mass of water, which seems to be arrested by a bank, considerably beneath its level. Ought this phenomenon to be attributed to mirage, *or are the waters really elevated towards the equator, and kept in their state of congestion by particular laws?*" 190.

The therapeutics of the Chevalier are in accordance with his medical topography. On the first day of African fever, a pediluvium—a ptisan—a grain of emetic tartar. 2d day, a pediluvium—dilute hydrochloric acid for drink. 3d day, cream of tartar. 4th day, ditto repeated. When a remission takes place, (if it ever take place) then bark. But of this enough!

---

8. *Preservation of the Stomach by Poison.* A man of the name of Patect, was executed for murder, in the Pays Bas, on the 27th of August last. Before punishment, he confessed that he was privy to the crime of poisoning, committed on his uncle by the surviving widow. The uncle had died on the 28th May of the year preceding—the body having been buried nearly sixteen months. Exhumation was ordered, and, notwithstanding the putrefaction of the whole of the body, the stomach was in such a state of preservation that the poisonous substance was detected in it. The journal in which this information is contained, does not give any farther particulars; but there can be little doubt, that the preservation of the stomach was owing to the poisonous substance, (most probably a mineral one,) contained in it. The chlorures, for example, have great power in preserving animal substances from putrefaction.—*Gazette de Santé.*

---

9. *Pathology of a Pinch of Snuff.** Who will now complain that the French outstrip us in minute pathology? Dr. Kinglake has rescued the profession on this side of the Channel from the obloquy which has sometimes been thrown on them in this respect. He has dissected a pinch of snuff most minutely, and proved, beyond all cavil or doubt, that—"the practice of snuff-taking is, *perhaps,* the most baneful that popular custom and familiarity have sanctioned as innoxious and gratifying." Dr. Kinglake has discovered, that this deadly narcotic poison is carried into the stomach "by the act of strong *inhalation* through the nostrils," where it must produce the same effects as henbane, wolfsbane, deadly nightshade, laurel, &c. only in a slow and insidious manner. "Loss of appetite," says Dr. K. "distressing sickness, gastric oppression, præcordial anxiety, acetous fermentation, flatulent distention, and deadly languor, are amongst the direct effects of admitting snuff into the stomach." And again, he exclaims—"What vital function can preserve its healthy state amidst such overwhelming affection of gastric excitability?" We answer—none, none! Dr. Kinglake's reasoning is demonstration itself; and although we see none of the effects above described among our snuff-taking neighbours, we most willingly grant, that they *ought to take place,* according to the best systems of pharmacology— and it is a vile shame that they *do not* take place accordingly. Whether it is that this deadly narcotic does not find its way, *by inhalation,* into the sto-

* Dr. Kinglake. Med. and Phys. Journ. December.

mach—or that, like some other substances, (as ipecacuan for instance,) it effects, in minute doses, are totally different from those that result from large ones—but so it is, that the snuff-takers of our acquaintance, eat, drink, and sleep, like other people—and, which is the *argumentum ad hominem*, consult us no more frequently for gastric affections, than those who never inhale a pinch of Fribourg or Lundifoot from June till January. This, we allow, is not as it *ought to be.*—But Dr. Kinglake may console himself with the reflection, that Sir Joseph Bankes experienced the same disappointment, when he boiled the fleas, and found that they did not (as they *ought*) turn red like lobsters.*

10. *Elements of Phrenology*: by George Combe, President of the Phrenological Society. Mr. George Combe, the well-known Phrenologist of Scotland, has just published a duodecimo, entitled "Elements of Phrenology." As far as we can judge, it presents an agreeable epitome of the science, and we recommend it to those who are desirous of knowing what phrenology means without bestowing any time upon the enquiry. The disinterested ardour of the phrenologist is strikingly shewn in the price of the work, for this is so low, that it is impossible for the author to be more than reimbursed should the whole impression be sold, as it undoubtedly will.

---

### X.

### BIBLIOGRAPHICAL RECORD;

* "Fleas are not lobsters—d——n their souls."—*Peter Pindar.*

